# Elevated Inflammatory Status and Increased Risk of Chronic Disease in Chronological Aging: Inflamm-aging or *Inflamm-inactivity*?

**DOI:** 10.14336/AD.2018.0326

**Published:** 2019-02-01

**Authors:** Michael G. Flynn, Melissa M. Markofski, Andres E. Carrillo

**Affiliations:** ^1^HCA South Atlantic Division, Charleston, SC 29492, USA; ^2^College of Charleston, Charleston, SC 29424, USA; ^3^University of Houston, Department of Health and Human Performance, Houston, TX 77204, USA; ^4^Department of Exercise Science, Chatham University, Pittsburgh, PA 15232, USA; ^5^FAME Laboratory, Department of Exercise Science, University of Thessaly, Trikala, Greece

**Keywords:** inflammation, exercise, physical activity, immune system, gerontology

## Abstract

Age-associated hyper-inflammation or “inflamm-aging” has been linked to the development of chronic diseases and characterized as an unavoidable aspect of aging. However, the inflamm-aging model does not adequately address the potential anti-inflammatory effects of exercise training and the potential for exercise to ameliorate several age-related diseases. In this brief review, we introduce a new paradigm—*inflamm-inactivity*—that describes a potent counter-measure to age-associated inflammatory illness.

Inflammation, an ancient mechanism for protection against injury and infection, is highly conserved and a critical aspect of our host defense [1]. Nevertheless, poorly regulated innate immunity and inflammation contribute to the development or maintenance of chronic disease, such that biomarkers of low-level chronic inflammation are now being recognized as important predictors of disease risk. Cardiovascular disease, type 2 diabetes, and osteoporosis, to name three, are known to be exacerbated by a poorly regulated innate immune system.

In 2000, Franceschi *et al*. [2] coined the term “inflamm-aging” to describe the elevated chronic inflammation commonly observed in older persons and the coincident increase in co-morbidities. It was suggested that inflamm-aging is “an inescapable result of the long lasting exposure to acute and chronic infections and the consequent lifelong antigenic burden” [3]. It is necessary to acknowledge that the introduction of this neologism [4] was a significant event that altered how we view the development of age-related disease. In subsequent writings Franceschi’s group recognized that hyper-inflammation alone was not sufficient to increase age-related disease and proposed that a “second hit” was required, such as a genetic pre-disposition to disease or an overly aggressive inflammatory response. The latter point and the ability of exercise training to significantly reduce inflammation, and reduce the likelihood of the “second hit,” is the foundation of our paper.

Although it seems counter-intuitive, there is an exercise training-induced anti-inflammatory effect, observed in both younger and older subjects [5-8]. There is some disagreement in the literature, yet an increasing volume of evidence points to the ability of regular, planned exercise, or a high level of physical activity, to reduce the inflammatory burden [9-11]. Gleeson *et al*. published a review in 2011 that comprehensively described the general mechanisms by which exercise exerts its anti-inflammatory actions, including a reduction in toll-like receptor (TLR) expression on monocytes and phenotypic switching of macrophages within adipose tissue, just to name a few [12]. We found that 10-12 weeks of moderate exercise lowered inflammatory biomarkers and monocyte TLR4 expression of previously sedentary, elderly subjects to similar levels as young subjects. These accumulating findings lead us to argue that the hyper-inflammatory state in older subjects is strongly linked to physical activity, is reversible, and may not be an inescapable part of the aging process [6]. In this brief review, we will present evidence in support of a newly-coined phrase ‘*inflamm-inactivity’* along with which we propose that a substantial proportion of the so-called age-associated hyper-inflammatory state is linked to a sedentary lifestyle and its subsequent impact on body fat accumulation.

## Inflamm-Aging

In 2000, Franceschi *et al*. [2] melded the concomitant occurrence of an increased risk of age-associated chronic diseases and the age-associated increase in pro-inflammatory cytokines into a new paradigm—inflamm-aging. Among the key tenets of their paradigm is that inflammatory cytokines played a key role in the aging process, influenced disease risk, and reduced life span. In their initial model, Franceschi *et al*. used the network theory of aging [2, 13, 14] to identify a cellular defense network that included oxygen free-radical scavenging, DNA damage repair, heat shock response, and UV-stress recovery. These systems, they contended, work together to protect the cell from aging [13]. They extended the network theory of aging by suggesting that disruption of the protective network is combined with a progressive pro-inflammatory surge, which they called inflamm-aging and argued that it is concomitant with increased disease risk, morbidity, and mortality. Their logic flows from a patient’s obligatory, lifelong exposure to, and defense against, antigens and a shift in the functional capacities of the immune system as we age—an antagonistic pleiotropy of debilitating cytokines and aberrant responses. That is, inflammatory responses that prevent infection in early life, contribute to disease in later life. In this early work, Franceschi *et al*. (2001) implicated the innate immune system as a primary culprit with a special focus on the macrophage and its propensity to produce inflammatory cytokines. In subsequent work, Franceschi’s group [3] included an anti-inflammaging paradigm. Specifically, that cortisol, transforming growth factor (TGF) beta, and other anti-inflammatory proteins work in opposition to the hyper-inflammatory state, increase longevity, and lead to successful aging. Giunta [15] discussed the anti-inflammaging paradigm and speculated that the hyper-activation of the hypothalmic-pituitary-adrenal axis with aging—and the resultant hyper-cortisolism—likely accelerates the slide into frailty through its negative influence on muscle, bone, adipose tissue, exercise capacity, metabolism, mood, and cognitive function. Thus, anti-inflammaging as defined by Giunta [15], is not an effective counter-measure, but instead contributes to a progressive decline in function that can lead to frailty in inactive older people.

Franceschi et al. [3, 16] listed and described several chronic diseases, such as cardiovascular disease and type 2 diabetes that could be linked to the late-life cytokine surge. However, the potential influence of physical activity on both inflammation and chronic diseases is conspicuous by its absence in the early work by this group. In 2014, Franceschi’s group acknowledged that exercise may be an effective counter measure for inflamm-aging [17]. Minciullo *et al*. [18] argued that if inflamm-aging helps us to explain and understand aging, anti-inflammaging is vital to longevity. Thus, the primary purpose of our paper is to highlight the potential of regular, planned exercise or high levels of physical or obligate activity to attenuate the hyper-inflammatory state commonly associated with aging.

Franceschi [3] used an evolutionary-based systems argument to make his group’s case. That is, the immune system, shaped by evolution, performs quite well in young people, but is inefficient and hyper-stimulated when asked to respond to the “evolutionary unpredicted” exposure over the succeeding decades [3]. Booth *et al*. [19] opined that the human genome evolved to be active, to find food, and to store energy efficiently in times of plenty so that it would be available in times of insufficiency. They argued that modern man’s ready access to food in large quantities and enforced inactivity result in a sedentary phenotype that leads to impaired metabolism, dysfunction, and chronic disease. Our task is to reconcile these elegant hypotheses and attempt to determine what proportion of inflamm-aging is attributable to the sedentariness that increases as we age—*inflamm-inactivity*.

Most of the chronic diseases associated with inflamm-aging are known to be positively influenced by physical activity. As an example, a recent work by Pinti *et al*. [20] details the link between inflamm-aging and the danger signal created by circulating mitochondrial DNA. The impact of sedentary versus an active lifestyle on mitochondrial function is well documented and provides an example of the multitude of positive effects exerted by regular exercise participation. In addition, we see evidence in the literature, and in our own work, that later life increases in inflammation may not be inescapable, but rather a result of low physical activity and/or high levels of body fat [6, 8, 10, 21-28]. It is not our intent to impugn the important work and intriguing hypothesis set forth by Franceschi and colleagues; rather, we hope to have physical activity considered as part of the inflamm-aging model. Are physically active individuals able to delay or prevent inflamm-aging? Does physical activity or planned exercise exert its beneficial effect on chronic disease by blunting inflammation? Is inflamm-aging escapable?

### Potential peril of excluding physical activity from the inflamm-aging model

We argue that leaving the exercise training influences out of the inflamm-aging model is at best incomplete and at worst attributing the hyper-inflammatory state to the influence of aging per se and not allowing for the substantial impact of exercise training on inflammation that we and others have reported in older and younger subjects [6, 23, 24, 29-31]. In fact, when considering inflammatory biomarkers, there are frequent examples in the literature of physically active older adults being indistinguishable from physically active young people [6, 29].

Franceschi *et al*. [3] described four potential protective mechanisms: molecular, cellular, systemic, and organismal. At the molecular level are DNA repair mechanisms, heat shock proteins, protein and organelle turnover, and anti-oxidant enzymes. At the cellular level, Franceschi and coworkers [3] list apoptosis, phagocytosis, cell senescence, and progenitor cell replacement of dead or damaged cells. At the systemic level are the immune, inflammatory, stress and neuroendocrine responses, and at the organismal level are behavioral/avoidance responses that could reduce damage or danger. Franceschi *et al*. [3] suggested that these responses, collectively, contribute to the survival and longevity of an organism; however, a strong case can be made to show a significant influence of exercise training, and associated lifestyle behaviors, in each of these four areas.

The largely positive effects of exercise training on chronic disease, physical, and mental health have been clearly documented. In addition, the potential for exercise training to influence function at the organismal, cellular, molecular and signaling levels is clear. Exercise training lowers inflammation [6-8, 24], increases heat shock protein transcription/protection [32], refines processes of immune function and apoptosis [32-36], improves anti-oxidant capacity [37], responsiveness to vaccine [38], wound healing [39], cell signaling [32, 40] the neuroendocrine response [41], skeletal muscle function [42], retards cellular senescence [33], and enhances cognitive function [43, 44]. Thus, the broad influence of exercise training on physiological systems and functions is suggestive of a significant influence on the aging process. While the present paper is focused on the potential anti-inflammatory influences of exercise training, the related influences of exercise training on damage mechanisms cannot be overlooked.

## Age-Associated Changes in Physical Activity

Participation in regular physical activity exerts anti-inflammatory effects that counter the age-associated increase in pro-inflammatory biomarkers [23]. Habitual physical activity, however, is highly variable throughout the course of a lifetime [45, 46]. Could age-associated variations in inflammatory biomarkers reflect age-associated variations in physical activity? Several researchers provided support for the contention that the frequency and intensity of physical activity declines with advancing age [45-48].

Despite the general acceptance of the importance of regular physical activity, physical inactivity is widespread. For example, less than half of adult men and women in the United States perform the recommended amounts of daily exercise, with levels of inactivity showing a steady rise with advancing age [45]. Xue *et al*. [48] 2012 followed physical activity patterns of 433 older women (70 - 79 years at baseline) for 12 years. The physical inactivity prevalence at baseline (48%) increased to 62% by the end of the study. Using accelerometer data, Troiano and colleagues reported an age-related decline in physical activity with <5% of individuals over 60 years of age meeting the physical activity recommendations [49]. These age-associated physical activity declines have important clinical implications that may be related to increased inflammation and chronic disease risk.

Along with the decline in physical activity participation, there appears to be an age-associated shift in the type/intensity of physical activity [47]. DiPietro *et al*. 2001 [47] described an age-associated decline in high-intensity physical activity participation that paralleled an increasing prevalence of physical inactivity. Recently, In and So [50] reported that only 1.2% of ~1400 older individuals (>65 years) participated in high-intensity exercise, compared to 7.4% of adults (19 - 64 years) and 11.5% of adolescents (<18 years). Conversely, 17.7% of elderly individuals participated in low-intensity exercise, compared to 8.2% of adults, and 2.5% of adolescents [50]. An age-associated decline in exercise intensity reflects the type of activities reported in different age groups. Specifically, common activities of younger, physically active individuals include higher intensity activities such as running, team sports, weight lifting, and aerobics [47]. The most prevalent activities reported among those >65 years of age are lower intensity, such as walking, gardening, golf using a cart, and home exercises [47, 51].

Exercise intensity differences within a physically active population are relevant when considering the anti-inflammatory effects of exercise, as demonstrated by the National Health and Nutrition Examination Survey III (1988 - 1994). Specifically, in 13,748 adult participants within the United States, the age-adjusted odds ratios for elevated CRP concentrations were 0.78, 0.59, and 0.30, for light, moderate, and vigorously active participants, respectively [52]. In contrast, Onambele-Pearson *et al*. 2010 [53] had 39 older volunteers (~73 years of age) complete either a low- or high-intensity 12-week resistance training intervention. The high-intensity group experienced significant strength gains compared to the low-intensity group, but a significant decrease in TNFα was reported only in the low-intensity group [53]. It is possible that a variety of exercise intensities may be beneficial in stimulating a potent anti-inflammatory effect.

Regardless of intensity, participation in physical activities provides health benefits that include anti-inflammatory actions. The age-associated decline in overall physical activity participation is of major concern for the progression of inflammation among older populations. The inflammatory consequences of a sedentary lifestyle will be described in a later section; but based on the research described above, age-associated changes in physical activity support the nascent notions proposed by our *inflamm-inactivity* paradigm.

## Inflammation and Physical Activity Status

Biomarkers of inflammation are consistently linked to chronic disease risk. For example, high levels of IL-6 and TNFα are associated with an increased risk of cardiovascular disease [54]. Additionally, high CRP and IL-6 concentrations are associated with type 2 diabetes and impaired glucose tolerance [55]. Consistent elevation of these biomarkers is associated with aging and an increase in chronic disease risk, but there are also modifiable characteristics associated with a lower pro-inflammatory biomarker concentration and disease risk. Two of these modifiable factors are physical activity and body composition.

### Inflammation and inactivity

Across the lifespan, physical activity is associated with a decreased and increased concentration of inflammatory and anti-inflammatory biomarkers, respectively. Several researchers reported a relationship between physical activity and inflammation [52, 56-61]. Physically active young and older adults had significantly lower concentrations of circulating pro-inflammatory biomarkers compared to young and older physically inactive adults [6, 23]. Moreover, 12-weeks of exercise training significantly decreased these biomarkers, obfuscating differences between physically active subjects and the previously physically inactive subjects [6]. In addition to exercise decreasing inflammatory biomarkers, highly conditioned older adults have elevated concentrations of anti-inflammatory biomarkers such as IL-10 [62].

In light of the suggestion that inflammation is an inescapable part of aging, research on children and adolescents builds a case for inactivity being responsible for elevated inflammatory biomarkers. For example, CRP is inversely related to physical fitness in children [59]. This relationship is also present in middle-aged [52] and older [61] adults. The inverse relationship between activity and inflammation is present in children as young as ten years old [63]. Finally, there is a similar correlation between body weight and inflammatory biomarkers that may partially explain higher inflammation in inactive people. Irrespective of age, overweight and obese individuals have increased inflammation [63-66].

## Physiological Changes Resulting from Inactivity

A cross-sectional comparison of physically inactive versus physically active subjects yields several significant differences such as body weight or fat mass differences. Other differences, such as metabolism and insulin signaling, are not as consistent. We believe this ambiguity is due to the interrelatedness of body composition, inflammation, and metabolism.

### Body Composition

Participation in regular exercise aids in body weight maintenance [67, 68]. For example, in studies comparing age-matched physically inactive and physically active adults, physically active subjects had significantly lower body weight [6, 24, 69]. The body weight difference can be considered a potential covariate. However, since inactivity likely contributed to a significant increase in body weight, it is difficult to separate the individual effects of inactivity and increased body weight. Regardless, increased body weight and body fat are consistently linked to higher inflammation [65, 70, 71].

An example of body composition having a systemic effect is exerted by the most abundant adipokine, adiponectin. Adiponectin has anti-inflammatory actions, including stimulating anti-inflammatory cytokine production and inhibiting NF-κB [72, 73]. In addition, adiponectin increases insulin secretion [74, 75] and may enhance fatty acid β-oxidation [75]. Although adiponectin is produced by adipocytes, more adipocytes do not equate to more adiponectin. Obese people have lower plasma adiponectin than non-obese people [76]. In an apparently paracrine action [72], the pro-inflammatory cytokines TNFα and IL-6, produced by adipocytes, inhibit adiponectin expression. Changes in adiponectin may be independent of weight change, as exercise training increases plasma adiponectin in older adults [77]. Collectively, this demonstrates a complex relationship between adiposity and inflammation and underscores the difficulty in separating inflammation resulting from age-associated body weight gain or physical inactivity.

### Insulin Resistance/Glucose Metabolism

Physical inactivity is consistently linked to elevated blood glucose and insulin resistance [56, 78, 79]. For example, five days of bedrest significantly increased fasting blood glucose, insulin, total cholesterol, and HOMA-IR (50%) in young adults [80], making bedrest an effective model to study inactivity-induced metabolic changes. Increases in fasting blood glucose and insulin resistance are important because these two variables are a component of metabolic syndrome—a condition that is also characterized by elevated inflammation [81, 82]. Furthermore, older adults are at an increased risk of developing metabolic syndrome and type 2 diabetes [83], but this risk is lower in physically active older adults [84]—suggesting that inactivity is a greater risk factor than age. Taken together, physically inactive individuals have higher amounts of inflammation, and this inflammation directly contributes to insulin resistance and type 2 diabetes [85, 86].

## Does Exercise Exert Anti-Inflammatory Actions?

As described above, high levels of physical activity, including aerobic exercise and resistance training, are linked to anti-inflammatory effects. Although there are numerous benefits to exercise, most of the research involving physical activity in relation to chronic, low grade inflammation resulted in an anti-inflammatory effect [87, 88]. Several mechanisms are involved in reducing chronic inflammation; with inflammation lowering effects of exercise training on the muscle, endothelial cells, and immune cells reported [89].

Conversely, researchers have reported no change in inflammation biomarkers as a result of regular exercise training [90, 91]. CRP levels were unchanged after 421 overweight or obese females with elevated systolic blood pressure were separated into either a control or one of three exercise groups for 6 months [90]. In another study involving overweight or obese patients (n=52) there were no differences in CRP levels between a control group of three-months exercise training group [91]. It is possible that a focus on additional inflammatory biomarkers may have yielded a different result; however, it’s clear that not all researchers have found an anti-inflammatory response to exercise. It is also possible the subjects’ overweight or obese status could have influenced the ability of exercise to exert an anti-inflammatory effect.

## Inflamm-Aging and The Physical Activity Habits of Centenarians

Centenarians provide a unique opportunity for further study and discovery of clues that may help counter inflamm-aging. For example, centenarians express an unusual combination of both pro- and anti-inflammatory biomarkers [3]. This cytokine balance may be a critical component of successful aging among this unique population. Specifically, centenarians appear to counteract the progression of inflamm-aging with potent anti-inflammatory activity that is not necessarily evident in the general population [3]. To delay inflamm-aging and promote successful aging, a number of anti-inflammaging strategies have been proposed, such as the development of safe, anti-inflammatory medications. Physical activity, however, exerts anti-inflammatory effects in both young and older individuals [23]. Physical activity participation is an important component of lifestyle that works in combination with genetics to dictate aging trajectories. Vigorous physical activity among an older population has been associated with a lower prevalence of multi-morbidities that may develop, at least in part, due to inflammatory processes [92]. Among a population of 878 centenarians studied in Chongqing, China, 64% had the physical abilities to live and function autonomously. Adequate physical exercise was described as a key lifestyle factor for this successfully aged population [93]. In another study, regular physical exercise was described among 1907 Japanese centenarians as a critical determinant of autonomy and successful aging [94]. The anti-inflammatory effects of physical activity and its importance as a component in establishing autonomous living suggests that regular physical activity may contribute to establishing a healthy balance of pro- and anti-inflammatory biomarkers that may delay the progression of inflamm-aging and its related morbidities in this unique population.

## Inflamm-Inactivity

New developments, in this case the impact of physical activity on inflamm-aging, require a new perspective. In this brief review, we introduced the term ‘*inflamm-inactivity’* that represents a proportion of the age-associated hyper-inflammatory state that occurs due to a sedentary lifestyle. Introducing *inflamm-inactivity* into the aging vernacular will allow for a clearer understanding of the development of a hyper-inflammatory state—an understanding that hyper-inflammation is multi-factorial and modifiable, with application of planned exercise or increased physical activity. Expanding the concepts that represent factors influencing the complex nature of age-associated inflammation can lead to more sophisticated prevention strategies. Specifically, an awareness of the impact a sedentary lifestyle has on aging (i.e., *inflamm-inactivity*) provides a new perspective that can lead to refined anti-inflammatory interventions strategically modified across the lifespan.

## Summary and Conclusions

We presented a new paradigm—*inflamm-inactivity*—to account for the significant proportion of the age-related hyper-inflammatory state due to a sedentary lifestyle. We reported that physical activity participation and intensity decline with advancing age; that this decline is likely a contributing factor in increasing inflammatory biomarkers; and that there are a host of diseases and metabolic disorders linked to the hyper-inflammatory state, also a key tenet of the inflamm-aging paradigm. These paradigms diverge at the contention that the hyper-inflammatory state is an inescapable consequence of advancing age. Exercise training and increased physical activity are consistently shown to be an effective counter-measure against hyper-inflammation. Additional research is required to evaluate the durability of the exercise counter-measure, the specific effects on aging and longevity, and nuanced conditions under which the anti-inflammatory effects of exercise are permitted. It should not be overlooked that exercise has positive influences on many of the chronic diseases identified in the inflamm-aging paradigm. It will take some time before the consequences of aging per se on inflammation and illness can be partitioned from the consequences of a sedentary lifestyle. We have provided evidence in this document that shows exercise training-induced, short-term reversal of elevated inflammatory biomarkers, receptors and cellular responses. It is not known how durable and sustainable these effects are but the age-associated “inflammation curve” is certainly shifted to the right in those who exercise regularly.
